# How do urbanization and alien species affect the plant taxonomic, functional, and phylogenetic diversity in different types of urban green areas?

**DOI:** 10.1007/s11356-023-28808-y

**Published:** 2023-07-25

**Authors:** Łukasz Dylewski, Weronika Banaszak-Cibicka, Łukasz Maćkowiak, Marcin K. Dyderski

**Affiliations:** 1 Department of Zoology, Poznań University of Life Sciences, Wojska Polskiego 71C, 60-625 Poznań, Poland; 2 Dmowskiego 81, 60-204 Poznań, Poland; 3 Institute of Dendrology, Polish Academy of Sciences, Parkowa 5, 62-035 Kórnik, Poland

**Keywords:** Urban biotic homogenization, Urbanization, Vegetation, Urban greenery, Alpha diversity, Beta diversity, Human pressure, Alien plant species

## Abstract

Human pressure on urban landscapes has serious consequences for urban plant species. Therefore, environmental and anthropogenic factors affect the assembly of urban wildlife in plant communities. For biodiversity conservation and ecosystem services in urban areas, it is crucial to understand the impacts of urbanization as well as the introduction of alien plant species on urban plant communities. On 47 sites in Poznań (W Poland), we studied variation within and between three management greenery habitats, i.e., urban parks, greenery associated with housing estates, and urban grasslands, as they relate to taxonomical, functional, and phylogenetic alpha and beta diversity. We also examined how urbanization (measured by ISA) and alien plant species relate to vegetation compositional differences. We found that both urbanization and alien plant species cover decreased alpha diversity, while urbanization had various impacts on beta diversity within each studied habitat. Our results suggest that human pressure leads to similarities in the urban flora, where plant species with specific functional traits adapted to the urban environment. To achieve sustainable urbanization, urban planners should not only create diverse green spaces but also eliminate alien plants, increasing the role of urban land management in promoting the wildness of plant biodiversity in cities.

**Supplementary Information:**

The online version contains supplementary material available at 10.1007/s11356-023-28808-y.

## Introduction

Urbanization can drastically alter both biotic and abiotic components of the environment (McKinney 2006). Urban landscapes differ from natural or semi-natural habitats. Human pressure in the urban landscape causes changes in the environment due to replacing the natural habitats with industrial and built-up areas and involves habitat fragmentation as well as creating novel biotic elements, i.e., managed green areas affect the ecosystem functioning and the species pool (Kowarik 2011). That way, urban conditions tend to promote generalist species instead of species with narrow ecological niches, e.g. woodland specialists (Kowarik 2011; Jarošík et al. 2011a; Dyderski et al. 2017a). However, the environmental structure may severely differ where some parts of the cities are more urbanized, i.e., by high-density of built-up areas, than others. Moreover, the other aspects of urbanization also affect urban ecosystems, especially light and soil pollution (Grimm et al. 2008; Russo and Ancillotto 2015). Consequently, the human pressure in the city can be easily measured by the impervious surface area, which is a good proxy of the urbanization level in the environment (Szulkin et al. 2020).

Understanding how urbanization processes affect the taxonomical, functional, and phylogenetic diversity of local plants and animals is a keystone for biodiversity conservation and the provision of ecosystem services in urban ecosystems (Olden et al. 2018). Functional traits are associated with the physiological, morphological, and phenological properties that determine the success of species in the communities (Violle et al. 2007). Therefore, functional traits affect reproduction, growth rate, and productivity, which may have important consequences for species surviving under human pressure in urban ecosystems (Czortek and Pielech 2020). Many plants and animals are well adapted to urban ecosystems, in particular, generalist species with high plasticity (Johnson and Munshi-South 2017). On the other hand, urbanization causes a direct decrease in species richness in highly urbanized areas and drives biological invasions, where many alien species over the years become more abundant and common in the urban habitat compared with the origin natural environment (Dyderski and Jagodziński 2016).

Rapid changes in the environment by increasing urbanization may filter the plants and animals which are better adapted to live in the city (Johnson and Munshi-South 2017), as well as opportunities for a new niche for alien species (Kowarik 2008; Knapp et al. 2010; Štajerová et al. 2017; Paź-Dyderska et al. 2020; Kowarik 2023) what may drive the high risk of biological invasions in the urban landscape. Biotic homogenization, considered a loss of compositional, functional, or phylogenetic distinctiveness among studied sites (Olden et al. 2004, 2018), is one of the most important threats to biodiversity (Winter et al. 2009; Thuiller et al. 2011; IPBES 2019). Cities, as hotspots of alien species introductions and vulnerable to the loss of specialized species, are therefore especially prone to biotic homogenization (McKinney 2006; Kühn and Klotz 2006; Lososová et al. 2016). This led to a loss of species dissimilarity among cities (La Sorte et al. 2014; Lososová et al. 2016). Biotic homogenization is well-recognized in urban ecosystems (Johnson et al. 2015). As similar habitats demand similar species characteristics, the urban biotic homogenization hypothesis proposes that such similarity of physical habitats will increase the similarity of species communities in cities (McKinney 2006). Recent studies confirm that urbanization leads to biotic homogenization within cities, meaning that the highly urbanized parts of a city are more similar in their species composition than less urban areas in or around the same city (Lokatis et al. 2023). However, functional and phylogenetic aspects of biotic homogenization within cities are significantly less recognized (Olden et al. 2018, Lokatis et al. 2023).

Many studies have shown that urban green spaces are critical for biodiversity conservation in the city. Human-transformed greenery (e.g., green roofs, urban parks, private gardens, and industrial patches), as well as remnant vegetation within a city (e.g., urban grasslands, wastelands, and woodlands), may be vitally important in preserving many species of plants and animals (Jarošík et al. 2011b; Dyderski et al. 2017b; Planchuelo et al. 2020). However, the management practice can modify the species composition and create biodiversity hotspots or coldspots in the urban landscape. For example, Dylewski et al. (2020) showed that vegetation composition around three habitat types is linked with pollinator composition. Thus, vegetation composition and structure are essential for the conservation values of animals, especially insects (Cross et al. 2016; Mata et al. 2017), amphibians (Hamer and McDonnell 2008), and birds (Threlfall et al. 2016) taxa in the city.

Urban ecosystems characterized by mosaic habitats with various managements of green areas transformed by human activity can be also associated with high plant species diversity, due to various management regimes (Chen et al. 2017; Planchuelo et al. 2020). The management practices like mowing, human trampling, clearing, and weeding will inhibit or alter succession processes and affect vegetation dynamics (Zipperer et al. 2011). These activities associated with urbanization alter the diversity, composition, and ground cover patterns of vegetation (Chen et al. 2017; Czortek and Pielech 2020; Planchuelo et al. 2020). Moreover, urbanization promotes the plant species with specific functional traits adapted to the specific abiotic environment (Dyderski et al. 2017a; Lososová et al. 2016; Czortek and Pielech 2020). For example, the city microclimate promotes the earlier flowering and zoochorous plant species (Czortek and Pielech 2020). However, the empirical evidence suggests that not all green spaces have an equal value for plant diversity (Jarošík et al. 2011a, 2011b; Dyderski et al. 2017a) where some greeneries are characterized by low or high-quality patches affected by the management practice and connected with the surrounding landscape. Moreover, the urbanization process involves decreasing the alpha and beta functional diversity and species turnover in the urban riparian forest, causing biotic homogenization in the plant communities. For example, Czortek and Pielech (2020) reported that plant functional diversity and dispersion were related to the surrounding landscape, where parks contribute to the high share of the settlements characterized by lower functional dispersion of plants and lower frequency of deciduous forest plant species. Consequently, environmental and anthropogenic filters acting on plant species traits affect the plant communities’ assembly of urban wildlife (Hu et al. 2022).

Here, we described variation within and between three different management greenery habitats, i.e., urban parks, greenery associated with housing estates (hereafter housing estate), and urban grasslands in three dimensions of biodiversity — taxonomic, functional, and phylogenetic in the urban landscape. Urban greenery in city habitat types is likely to be strongly affected by regional homogenization of the species pool but varies by management policy and environmental alternation. We compared community composition and levels of a- and b-diversity of herbaceous plants between urban parks, greenery around housing estates, and urban grasslands. We also assessed to what extent compositional difference metrics were explained by urbanization measured by impervious surface area (ISA), number of alien plant species, and cover of alien plant species.

We hypothesize that (1) urbanization affects the taxonomical, functional, and phylogenic diversity and (2) management practices in three habitat types affect the taxonomical, functional, and phylogenic plant diversity; (3) alien plant species have a negative effect on dimensions of biodiversity (taxonomical, functional, and phylogenic diversity). We predicted that urbanization and alien plant species would decrease taxonomical, functional, and phylogenic plant diversity in the urban landscape.

## Materials and methods

### Study area

We conducted our study in the city of Poznań (West Poland; 52°24′N, 16°57′E). Poznań is characterized by various land-use types, dominated mainly by built-up areas 41% and arable land 22%. Urban green spaces, including forests, comprise 27% (Jackowiak 2011). In Poznań, we selected 47 sites, each with approximate dimensions of 250 × 250 m, in three different urban habitat types: urban parks (13 sites), housing estates (22 sites), and urban meadows (12 sites). We defined urban parks as green areas designed for human recreation with intensive management practices (e.g., frequent mowing and pruning of trees and shrubs). We defined housing estates as small (< 0.05 ha) or medium greenery patches (0.05–0.5 ha) between the apartment blocks, characterized by a low management regime to reduce cost by most housing companies and the public. Mainly that urban space was sown with grass and legume plant species, especially *Lolium perenne*, *Festuca rubra*, and *Trifolium repens.* Thus, these green areas are similar to lawns; however, they are mowed less often and are not fertilized. We defined urban grasslands as open, semi-natural green areas in the city, mostly in the river valley, characterized by the lack of management practices and comparable to semi-natural grasslands.

### Data collection

Vegetation sampling was carried out from June to August 2016. To compile a list of plant species and some habitat features, we created a total of 169 vegetation records (on 47 study sites). Three-four plots (25 m^2^) were established within each site to cover full vegetation heterogeneity and floristic diversity. Plant species abundance was determined once per study site at the peak of the vegetation season (i.e., from late July to early August) with the Braun-Blanquet scale, modified by Barkmann et al. (1964), i.e., < 1%, 1–5%, 6–15%, 16–25%, 26–50%, 51–75%, and 76–100%. All collected data were entered into the Turboveg program (Hennekens and Schaminée 2001). Additionally, we measured the height of the herbaceous vegetation (cm) in three places within each plot once a month (from June to August). We used the mean height of plants for each site (in total *n* = 27 measurements per site).

### Data analyses

We analyzed data using R software (R Development Core Team 2021). We used a phylogenetic tree for the dataset of species present in our plots from the megatree included in V.phylo.maker package (Jin and Qian 2019). We acquired species functional traits from LEDA (Kleyer et al. 2008), BIEN (Maitner et al. 2018), BiolFlor (Klotz et al. 2002), and Pladias (Chytrý et al. 2021) databases. We included three traits representing leaf economic spectrum and plant size: specific leaf area (SLA, cm^2^ g^−1^), plant height (H, m), and seed mass (SM, mg), following Cubino et al. (2021). Lastly, we included Ellenberg’s ecological indicator values (EIVs) as an approximation of species environmental requirements (Ellenberg and Leuschner 2010). As datasets were incomplete for some species, we imputed missing data rather than omit them in analyses (Pyšek et al. 2015). We used the random forest–based imputation (Penone et al. 2014) in the *missForest* package (Stekhoven and Bühlmann 2012), using known values of traits and phylogenetic eigenvectors (Diniz-Filho et al. 1998), obtained using the *PVR* package (Santos 2018). The first 15 phylogenetic eigenvectors covered 63.2% of the variation in phylogenetic distances among species. For three main traits (SLA, height, and seed mass) and Ellenberg’s ecological indicator values, we calculated community-weighted mean values (CWM), using cover as a weight.

We analyzed three aspects of plant species diversity — taxonomic, phylogenetic, and functional, at two levels — alpha (within-site) and beta (among sites). We calculated taxonomic alpha diversity using species richness and Shannon’s diversity index. We calculated functional diversity by functional richness (FRic), expressing the quantity of plant functional types present in a community; functional dispersion (FDis), expressing the size of community species traits hypervolume within the functional trait space; functional divergence (FDiv), expressing on how large the average distance of each species to the center of gravity (center-space) of the trait space; and functional evenness (FEve), informing about on the degree of evenness of the distribution of biomass in a niche space (Mason et al. 2005; Laliberté and Legendre 2010; Pla et al. 2011). These indices were calculated using the *FD* package (Laliberté et al. 2014). We quantified phylogenetic diversity using Faith’s phylogenetic diversity (PD; i.e., the sum of phylogenetic tree branch lengths, representing all species present in the community), mean nearest taxon distance (MNTD), and mean pairwise phylogenetic distance (MPD) between species within the community. We calculated them using the *PhyloMeasures* package (Tsirogiannis and Sandel 2016). Negative values of PD, MNTD, and MPD indicate strong phylogenetic clustering, i.e., a higher frequency of species representing particular clades than under random circumstances.

We calculated beta diversity indices using Jaccard’s dissimilarity index, as this metric was the most frequently used in previous studies on biotic homogenization (Olden et al. 2018). Taxonomic beta diversity was calculated using a species presence-absence matrix, functional diversity using the volume of convex hull intersections in a multidimensional functional space (extracted from principal coordinates analysis from species traits of a Gower dissimilarity matrix) while phylogenetic diversity using a matrix of phylogenetic distances. These indices were calculated using the *betapart* package (Baselga et al. 2018). For each beta diversity index, we calculated the overall value, nestedness, and turnover (Baselga 2010). This allowed us to explain the importance of nestedness (presence of core species) and turnover (species replacement) in shaping dissimilarities among particular vegetation types and studies.

We used impervious surface area (ISA) as a proxy for urbanization. In each site, we calculated ISA using a 20-m pixel resolution map of ISA obtained from satellite imagery from 2015 in a 250-m buffer around the centric point (Copernicus Land Monitoring Services, https://land.copernicus.eu/sitemap). We decided to use that buffer size to avoid spatial autocorrelation, connected with overlapping buffers from neighbor sites (Dylewski et al. 2019). All built-up areas, i.e., infrastructural networks and buildings, are included in this index. The index was calculated with QGIS (version 2.18.15) open access software.

We used generalized linear models to evaluate the effects of habitat type, urbanization, and alien species on all studied alpha diversity metrics. Within models, we treated alpha diversity metrics as a response while habitat type, urbanization, and alien species richness or cover as predictors. We tested both impacts of alien species richness and cover in alternative models, to evaluate both aspects of invasions. We added the quadratic term of the number of alien plants and the cover of alien plants in models where we found the non-linear relationship based on Akaike’s information criterion, corrected for small sample size (AICc). We also included two interactions between habitat type and urbanization and habitat type and alien species (number of alien species or cover of alien species).

Next, we used linear models to evaluate the effects of habitat type and urbanization differences as well as the interaction between them on all studied beta diversity metrics. A low value of urbanization differences means that two sites have similar urbanization levels, while a high value of urbanization differences means that two sites have different urbanization levels. Thus, sites within the cities with more highly urbanized areas could have a more similar plant species composition than sites with less urbanized areas.

Using Akaike’s information criterion for small sample sizes (AICc), we compared the models with interactions with models without interaction terms, to select the final models (with the lowest AICc). We also provided Cohen’s *f* statistic as a measure of effect size, for easier comparison of models.

We used a non-metric multidimensional scaling (NMDS) to assess the species composition of vegetation. We decided to use NMDS due to non-linear gradients in species composition. This ordination method was implemented in the vegan package (Oksanen et al 2018). We passively fit alpha diversity indicators, urbanization, alien species number and cover, and CWMs using the envfit function in the *vegan* package (Oksanen et al. 2018).

## Results

### Vegetation and environmental feature differences between habitat type

We found 266 plant species from 45 families: 159 species in grasslands, 191 in housing estates, and 136 in urban parks. We found that the vegetation height (*F*_2,44_ = 15.45, *p* < 0.001), as well as urbanization (*F*_2,44_ = 6.23, *p* = 0.003), significantly differed among habitat types. However, the number of alien plant species (χ^2^ = 1.16, *df* = 2, *p* = 0.559) and their cover (χ^2^ = 3.36, *df* = 2, *p* = 0.186) did not differ among habitat types. We also did not find any differences in CWMs of seed mass (*F*_2,44_ = 0.91, *p* = 0.410), SLA (*F*_2,44_ = 0.17, *p* = 0.847) and plant height (F_2,44_ = 0.72, *p* = 0.493), as well as in Ellenberg’s ecological indicator values for light (*F*_2,44_ = 0.33, *p* = 0.721), temperature (*F*_2,44_ = 0.22, *p* = 0.802), between three habitat types, except soil moisture (*F*_2,44_ = 5.81, *p* = 0.006), soil reaction (*F*_2,44_ = 3.35, *p* = 0.044), and soil fertility (*F*_2,44_ = 3.60, *p* = 0.036) (Fig. S1).

### Impact of urbanization and alien plant species on taxonomic, functional, and phylogenetic alpha diversity

**Fig. 1:**
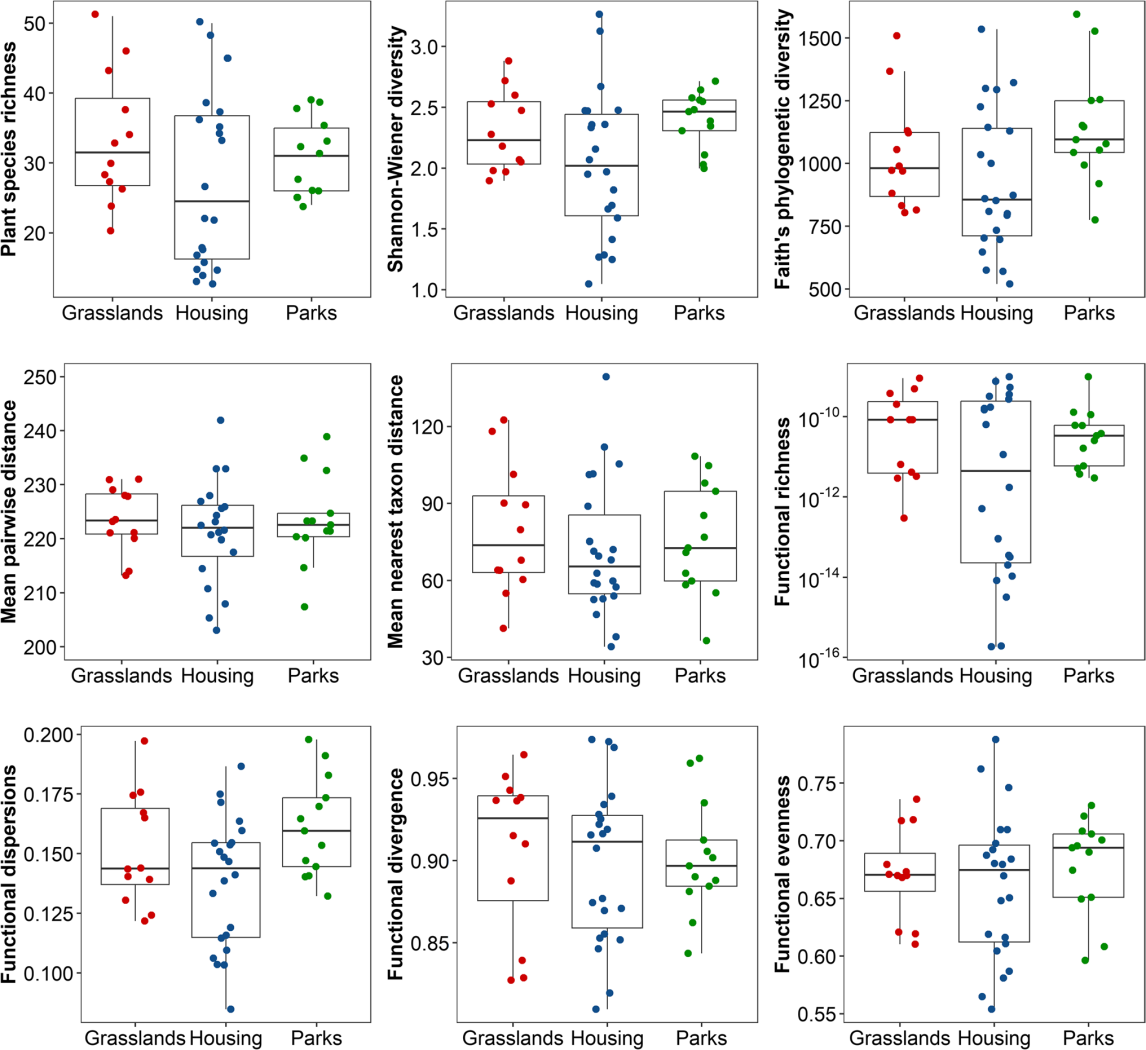
Boxplot of taxonomical, functional, and phylogenetic alpha diversity for plant species in urban parks, housing estates, and urban grasslands. The points represent study sites

**Fig. 2:**
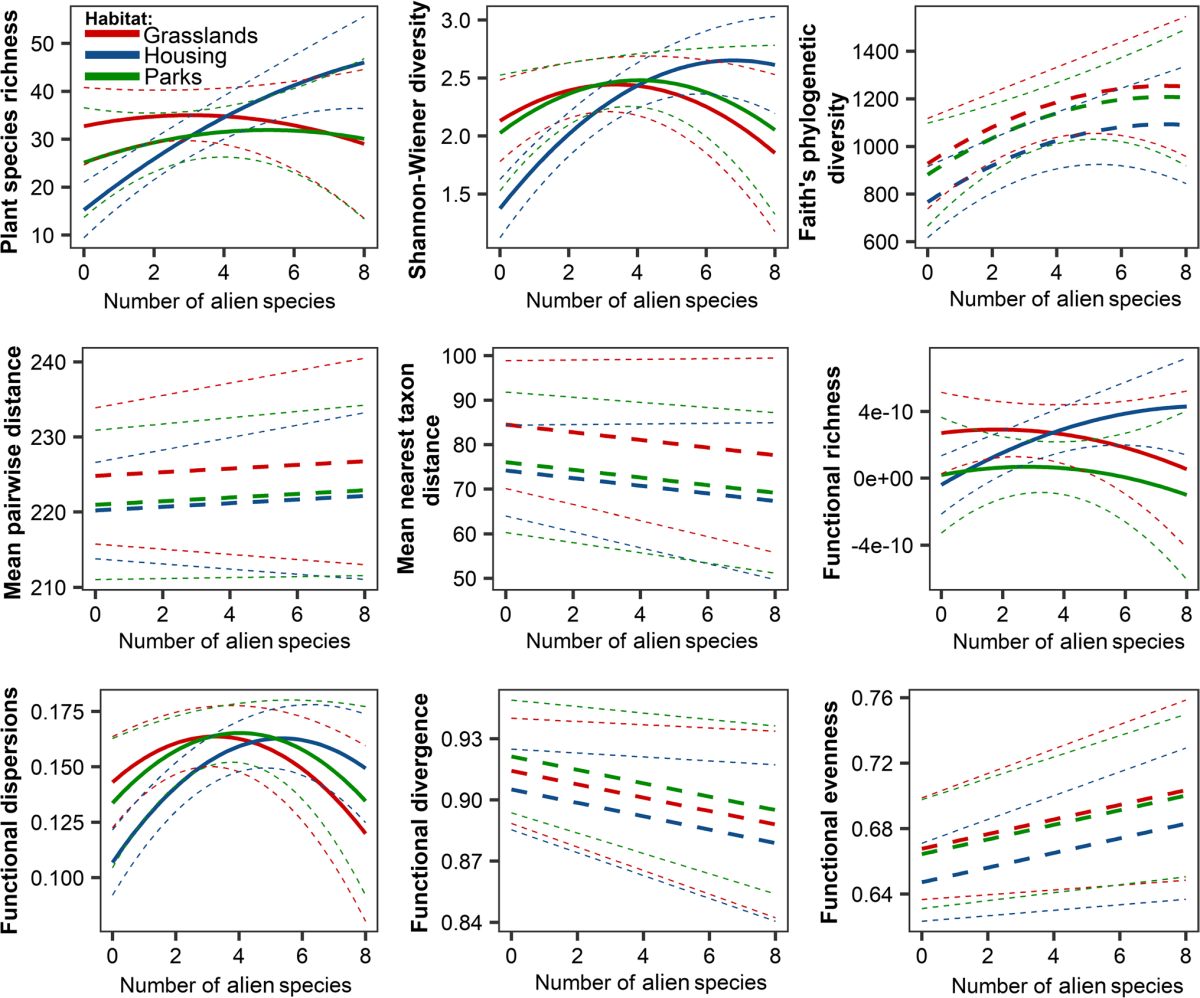
Effect of the number of alien plant on taxonomical, functional, and phylogenetic alpha diversity for plant species according to habitat type (Table 1). The solid line indicates significant interaction, while the dotted-point line indicates non-significant interaction. The dotted lines indicate 95% confidence intervals

In models that included the number of alien species as an explanatory variable, we found that urbanization had a significant positive effect only for phylogenetic diversity (PD) (estimate ± SE = 7.93 ± 3.72, *F*_1,44_ = 4.54, *p* = 0.039). In contrast, in the case of other alpha diversity metrics, it had no significant effect (*p* < 0.05, Tab. 1). The number of alien plant species had a significant nonlinear effect on Shannon–Wiener diversity (alien: 0.18 ± 0.09, *F*_1,39_ = 4.35, *p* = 0.044; alien^2^: − 0.03 ± 0.01, *F*_1,39_ = 6.06, *p* = 0.018) and functional dispersion (alien: 0.09 ± 0.04, *F* = 6.25, *p* = 0.017; alien^2^: − 0.01 ± 0.00, *F* = 9.17, *p* = 0.004). We found that taxonomic, functional, and phylogenetic alpha diversity were similar among the three habitat types studied (Fig. 1). Even though models showed that habitat types were significantly different in taxonomical, functional, and phylogenetic alpha diversity, the posteriori Tukey test did not report any significant differences between the urban parks, housing estates, and grasslands (Table 1). Our results showed the interaction term number of alien plants × habitat type had a significant non-linear effect on species richness (*F*_2,39_ = 5.84, *p* = 0.006, Fig. 2), Shannon–Wiener index (*F*_2,39_ = 6.14, *p* = 0.005, Fig. 2), functional richness (*F*_2,39_ = 5.74, *p* = 0.006: Fig. 2), and functional dispersion (*F*_2,39_ = 3.61, *p* = 0.037: Fig. 2).

**Table 1:** The results of the generalized linear models testing effect of the number of alien plant species (*alien*_*sp*_) and quadratic term: *alien*_*sp*_^2^, impervious surface area (ISA), habitat type, and interaction habitat type × ISA and habitat type × *alien*_*sp*_ on taxonomical, functional, and phylogenetic alpha diversity. The effect size is represented by Cohen’s *f* statistic

	Effect size	*F*	*df*	*p*
Species richness				
* alien*_*sp*_	0.71	0.54	1, 39	0.468
* alien*_*sp*_^2^	0.11	0.91	1, 39	0.347
ISA	0.06	0.33	1, 39	0.567
habitat type	0.35	7.49	2, 39	0.002
habitat type × *alien*_*sp*_	0.55	5.84	2, 39	0.006
Shannon’s diversity index				
* alien*_*sp*_	0.76	4.35	1, 39	0.044
* alien*_*sp*_^2^	0.43	6.06	1, 39	0.018
ISA	0.07	0.03	1, 39	0.872
habitat type	0.37	8.58	2, 39	0.001
habitat type × *alien*_*sp*_	0.56	6.14	2, 39	0.005
Faith’s phylogenetic diversity (PD)				
* alien*_*sp*_	0.82	2.79	1, 41	0.103
* alien*_*sp*_^2^	0.45	0.70	1, 41	0.408
ISA	0.33	4.54	1, 41	0.039
habitat type	0.32	2.06	2, 41	0.141
Mean pairwise phylogenetic distance (MPD)				
* alien*_*sp*_	0.02	0.05	1, 42	0.825
ISA	0.14	1.07	1, 42	0.305
habitat type	0.12	0.30	2, 42	0.740
Mean nearest taxon distance (MNTD)				
* alien*_*sp*_	0.10	0.25	1, 42	0.620
ISA	0.10	0.88	1, 42	0.352
habitat type	0.17	0.60	2, 42	0.554
Functional richness (FRic)				
* alien*_*sp*_	0.70	1.26	1, 39	0.267
* alien*_*sp*_^2^	0.24	1.64	1, 39	0.208
ISA	0.12	0.53	1, 39	0.469
habitat type	0.46	9.70	2, 39	< 0.001
habitat type × *alien*_*sp*_	0.54	5.74	2, 39	0.006
Functional dispersion (FDis)				
* alien*_*sp*_	0.50	6.25	1, 39	0.017
* alien*_*sp*_^2^	0.58	9.17	1, 39	0.004
ISA	0.20	0.81	1, 39	0.372
habitat type	0.35	6.24	2, 39	0.004
habitat type × *alien*_*sp*_	0.40	3.61	2, 39	0.037
Functional divergence (FDiv)				
* alien*_*sp*_	0.36	3.44	1, 41	0.071
* alien*_*sp*_^2^	0.25	2.01	1, 41	0.164
ISA	0.06	0.15	1, 41	0.699
habitat type	0.13	0.51	2, 41	0.606
Functional evenness (FEve)				
* alien*_*sp*_	0.10	0.91	1, 41	0.345
* alien*_*sp*_^2^	0.11	1.49	1, 41	0.229
ISA	0.23	1.44	1, 41	0.237
habitat type	0.17	0.69	2, 41	0.506

**Fig. 3:**
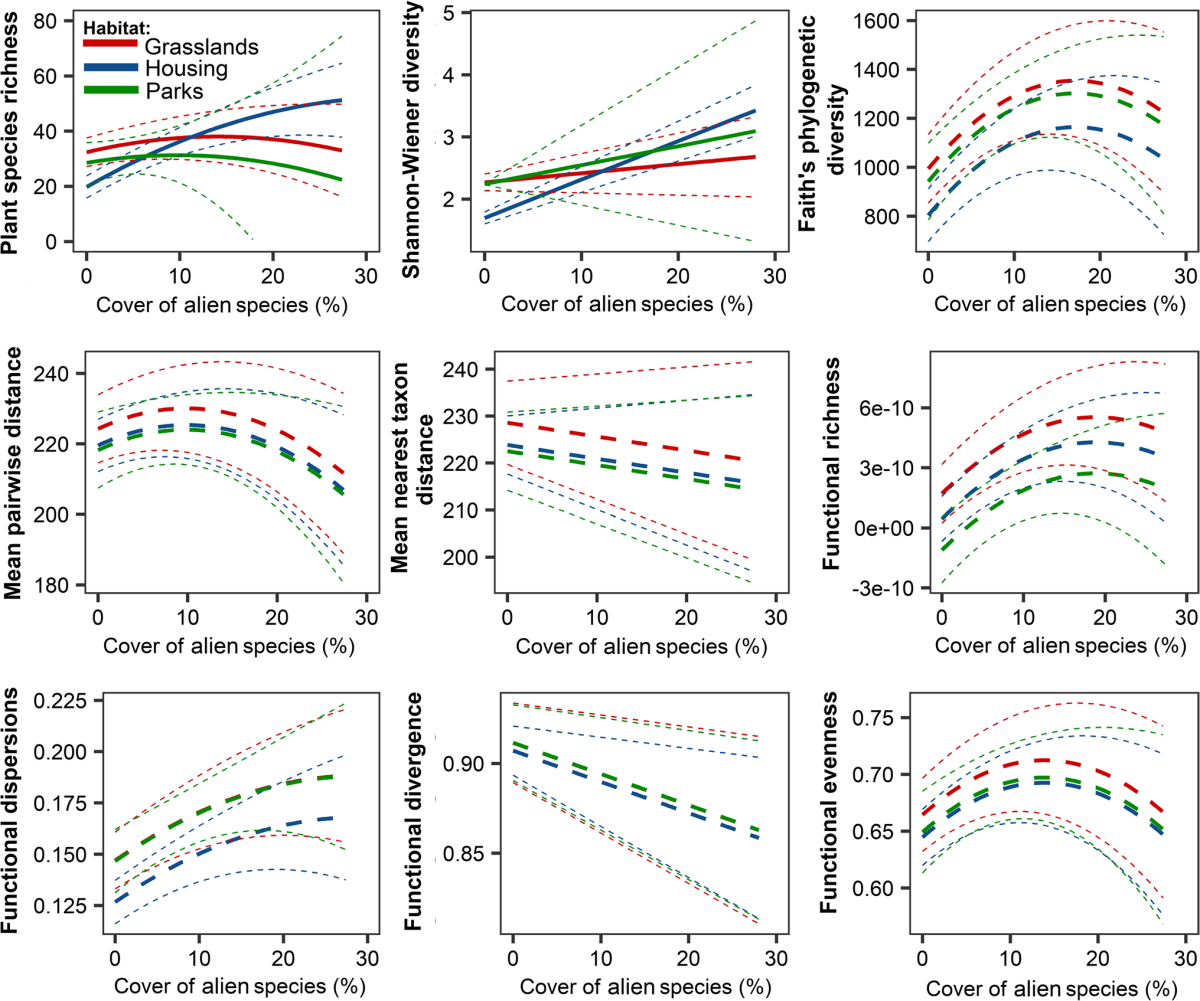
Effect of the cover of alien plant on taxonomical, functional, and phylogenetic alpha diversity for plant species according to habitat type (Table 2). The solid line indicates significant interaction, while the dotted-point line indicates non-significant interaction. The dotted lines indicate 95%confidencet intervals

In models including the cover of alien species as an explanatory variable, we found that urbanization had a significant positive effect only for phylogenetic diversity (PD) (8.90 ± 3.63, *F*_1,41_ = 6.02, *p* = 0.018). We also found that taxonomic, functional, and phylogenetic alpha diversity was similar among the three habitat types studied, evaluated by the posteriori Tukey test. Our result indicated that the interaction term cover of alien plant × habitat type was significant for taxonomical diversity (*species richness F*_2,39_ = 4.18, *p* = 0.023, Fig. 3; *Shannon–Wiener index*: *F*_2,40_ = 3.73, *p* = 0.032: Fig. 3).

**Table 2:** The results of the generalized linear models testing effect of the cover of alien plant species (*alien*_*cov*_) and quadratic term: *alien*_*cov*_^2^, impervious surface area (ISA), habitat type, and interaction habitat type × ISA and habitat type × *alien*_*cov*_ on taxonomical, functional, and phylogenetic alpha diversity. The effect size is represented by Cohen’s *f* statistic

Response	Effect size	*F*	*df*	*p*
Species richness				
* alien*_*cov*_	0.72	1.21	1, 39	0.278
* alien*_*cov*_^2^	0.26	1.34	1, 39	0.255
ISA	0.11	0.56	1, 39	0.459
Habitat type	0.41	6.80	2, 39	0.003
Habitat type × *alien*_*cov*_	0.46	4.18	2, 39	0.023
Shannon’s diversity index				
* alien*_*cov*_	0.86	1.04	1, 40	0.313
ISA	0.22	0.64	1, 40	0.430
Habitat type	0.59	8.92	2, 40	0.001
Habitat type × *alien*_*cov*_	0.43	3.73	2, 40	0.032
Faith’s phylogenetic diversity (PD)				
* alien*_*cov*_	0.34	7.59	1, 41	0.009
* alien*_*cov*_^2^	0.36	3.75	1, 41	0.059
ISA	0.39	6.02	1, 41	0.018
Habitat type	0.39	3.13	2, 41	0.054
Mean pairwise phylogenetic distance (MPD)				
* alien*_*cov*_	0.10	1.25	1, 41	0.270
* alien*_*cov*_^2^	0.20	1.86	1, 41	0.179
ISA	0.12	1.22	1, 41	0.275
Habitat type	0.14	0.41	2, 41	0.668
Mean nearest taxon distance (MNTD)				
* alien*_*cov*_	0.14	0.48	1, 42	0.494
ISA	0.09	0.86	1, 42	0.360
Habitat type	0.17	0.57	2, 42	0.569
Functional richness (FRic)				
* alien*_*cov*_	0.40	8.49	1,41	0.006
* alien*_*cov*_^2^	0.16	2.93	1,41	0.094
ISA	0.38	2.14	1,41	0.151
Habitat type	0.40	4.70	2,41	0.014
Functional dispersion (FDis)				
* alien*_*cov*_	0.44	3.57	1,41	0.066
* alien*_*cov*_^2^	0.20	0.6	1,41	0.438
ISA	0.31	3.13	1,41	0.083
Habitat type	0.48	4.67	2,41	0.015
Functional divergence (FDiv)				
* alien*_*cov*_	0.25	2.77	1,42	0.103
ISA	0.10	0.32	1,42	0.574
Habitat type	0.05	0.06	2,42	0.945
Functional evenness (FEve)				
* alien*_*cov*_	0.13	3.88	1,41	0.055
* alien*_*cov*_^2^	0.27	2.92	1,41	0.095
ISA	0.18	1.68	1,41	0.202
Habitat type	0.16	0.51	2,41	0.604

### Impact of urbanization on taxonomic, functional, and phylogenetic beta diversity

We indicated that difference in urbanization had a significant negative effect on taxonomic (*F*_1,744_ = 60.21, *p* < 0.0001) and phylogenetic (*F*_1,744_ = 25.98, *p* < 0.0001) but positive on functional (*F*_1,744_ = 25.13, *p* < 0.0001) overall beta diversity. We found a negatively significant relationship in taxonomical nestedness and turnover (*F*_1,744_ = 11.92, *p* = 0.0006; *F*_1,744_ = 61.01, *p* < 0.0001, respectively), functional nestedness (*F*_1,744_ = 5.61, *p* = 0.018), and phylogenetical turnover (*F*_1,744_ = 28.16, *p* < 0.0001) with urbanization difference, except functional turnover (*F*_1,744_ = 1.53, *p* = 0.216) and phylogenetic nestedness (*F*_1,744_ = 0.62, *p* = 0.429), where we did not find any significant relationship. Urban parks were characterized by lower taxonomical, functional, and phylogenetic beta diversity than urban grasslands and housing estates. Functional and phylogenetic beta diversity were similar in urban grasslands and housing estates; however, taxonomical diversity was higher in housing estates than in urban grasslands.

**Fig. 4:**
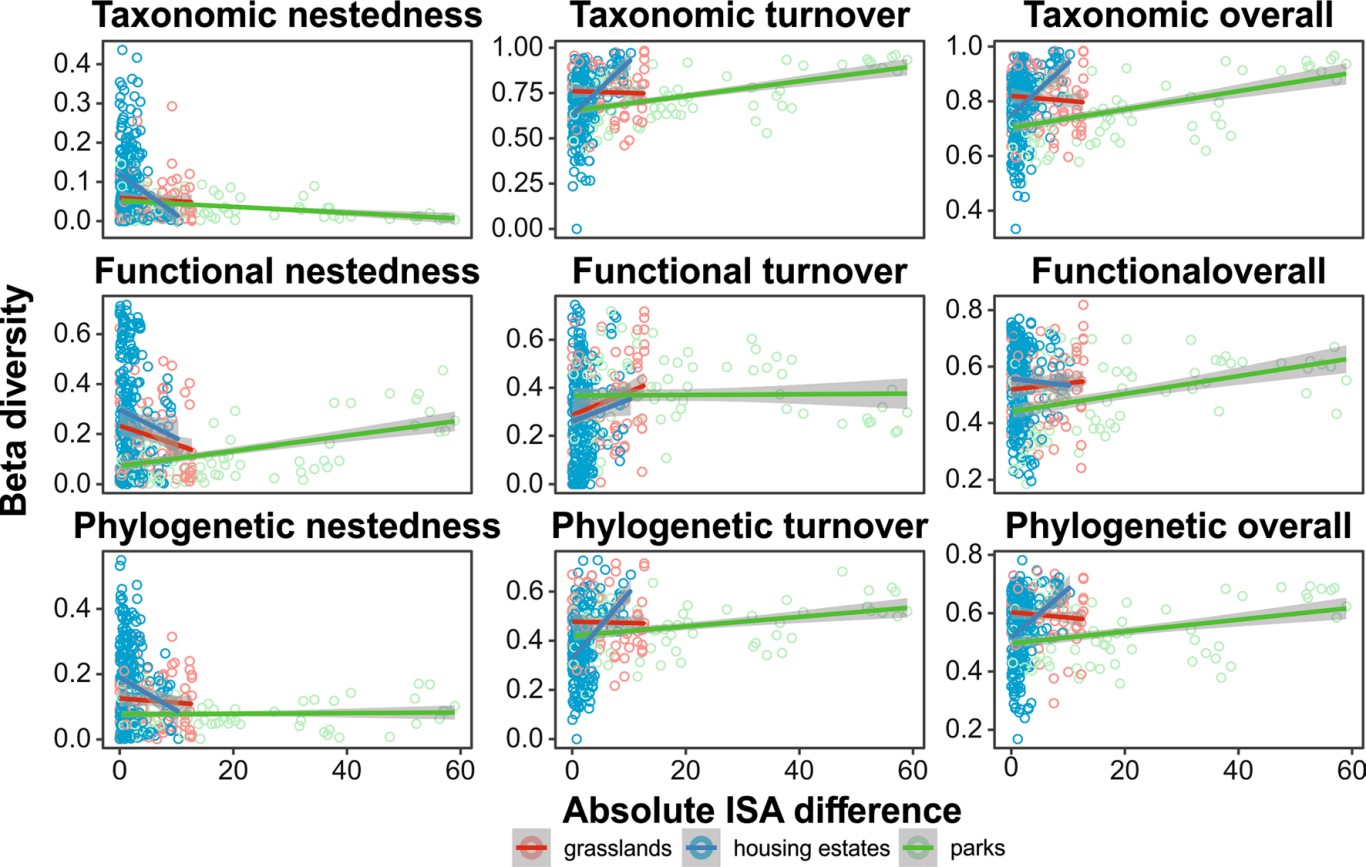
Effect of absolute ISA difference on taxonomical, functional, and phylogenetic nestedness, turnover, and overall beta diversity for plant species according to habitat type (Table 3)

We found biologically relevant interactions between urbanization differences and habitat type in all studied aspects of beta diversity (Table 3). We indicated different slopes in urbanization differences between habitat types on taxonomical, functional, and phylogenetic beta diversity (Fig. 4). In housing estates, the taxonomic and phylogenetic diversity increase with the difference in urbanization, but functional diversity decreases. The taxonomical and phylogenetic diversity in urban grasslands decreases with the difference in urbanization. In urban parks, the taxonomic, functional, and phylogenetic diversity increase with the difference in urbanization. We also observed that the taxonomic, functional, and phylogenetic nestedness of beta diversity in housing estates and grasslands decreased with the difference in urbanization, whereas functional nestedness in urban parks increased with the difference in urbanization. Taxonomical, functional, and phylogenetic turnover in housing estates increased with the difference in urbanization. In grasslands, we observed a positive trend with functional turnover but a negative trend in taxonomic and phylogenetic turnover. In urban parks, taxonomic and phylogenetic turnover increase with the difference in urbanization, except functional turnover, where the slope was constant.

**Table 3:** The results of the generalized linear models testing effect of habitat type and absolute difference in imperious surface area (*ISA*_*d*_) on taxonomical, functional, and phylogenetic beta diversity. The effect size is represented by Cohen’s *f*

Response	Effect size	*F*	*df*	*p*
Taxonomic nestedness				
Habitat type	0.35	45.50	2, 744	< 0.0001
* ISA*_*d*_	0.13	11.92	1, 744	0.001
Habitat type × *ISA*_*d*_	0.21	16.25	2, 744	< 0.0001
Taxonomic — turnover				
Habitat type	0.18	11.82	2, 744	< 0.0001
* ISA*_*d*_	0.29	61.00	1, 744	< 0.0001
Habitat type × *ISA*_*d*_	0.30	34.46	2, 744	< 0.0001
Taxonomic — overall				
Habitat type	0.14	7.05	2, 744	0.001
* ISA*_*d*_	0.28	60.21	1, 744	< 0.0001
Habitat type × *ISA*_*d*_	0.26	24.46	2, 744	< 0.0001
Functional — nestedness				
Habitat type	0.34	43.35	2, 744	< 0.0001
* ISA*_*d*_	0.09	5.61	1, 744	0.018
Habitat type × *ISA*_*d*_	0.16	9.94	2, 744	0.0001
Functional — turnover				
Habitat type	0.22	18.22	2, 744	< 0.0001
* ISA*_*d*_	0.05	1.53	1, 744	0.216
Habitat type × ISA_d_	0.12	5.68	2, 744	0.003
Functional — overall				
Habitat type	0.18	11.42	2, 744	< 0.0001
* ISA*_*d*_	0.18	25.13	1, 744	< 0.0001
Habitat type × *ISA*_*d*_	0.07	1.77	2, 744	0.1707
Phylogenetic — nestedness				
Habitat type	0.34	43.86	2, 744	< 0.0001
* ISA*_*d*_	0.03	0.62	1, 744	0.429
Habitat type × *ISA*_*d*_	0.15	8.70	2, 744	< 0.0001
Phylogenetic — turnover				
Habitat type	0.34	42.17	2, 744	< 0.0001
* ISA*_*d*_	0.20	28.16	1, 744	< 0.0001
Habitat type × *ISA*_*d*_	0.34	43.98	2, 744	< 0.0001
Phylogenetic — overall				
Habitat type	0.18	11.97	2, 744	< 0.0001
* ISA*_*d*_	0.19	25.98	1, 744	< 0.0001
Habitat type × *ISA*_*d*_	0.24	20.72	2, 744	< 0.0001

### Plant species composition in urban green area

**Fig. 5:**
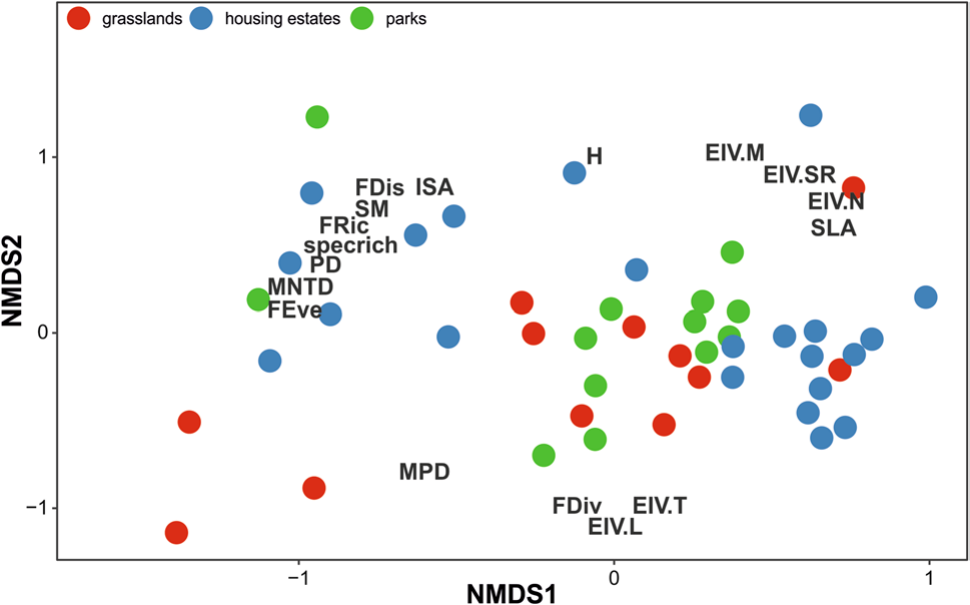
Non-metric multidimensional scaling of vegetation species composition (stress = 0.174). Points represent site scores, italic labels present scores of species with cover sum > 10% (e.g., *Festovin* = *Festuca ovina*), bold labels present passively fit vegetation characteristics (see Table S1)

NMDS revealed that points representing particular habitat types overlapped within the ordination space (Fig. 5). The main gradient (NMDS) was related to soil fertility, moisture, and reaction Ellenberg’s ecological indicator values and SLA CWM (positive NMDS1 values). Most alpha diversity indicators (species and functional richness, functional dispersion and evenness, PD, and MNTD) and seed mass CWM were negatively correlated with the NMDS1 gradient and positively with urbanization. The second gradient (NMDS2) differentiated between sites with high plant height CWM and sites with high MPD, functional divergence, and Ellenberg’s ecological indicator values of light and temperature.

## Discussion

Our results support the hypothesis that urbanization lead to taxonomical, functional, and phylogenetic homogenization of plant diversity within cities, which is consistent with the literature (McKinney 2006; Lososová et al. 2016; Trentanovi et al. 2013). Although the level of urbanization significantly differed between the three habitat types, we did not find differences in the plant species richness among the habitats studied. Moreover, the plant taxonomic, functional, and phylogenetic alpha diversity were similar in housing estates, urban parks, and urban grasslands. These results suggest that urbanization filters plant species from the species pool, which causes the displacement of rare species by those better adapted to the urban environment, including non-native plants. Furthermore, urban greenery is characterized by high disturbance by humans, which increases competition between species (Harrison and Winfree 2015; Fukano et al. 2022). We found that urbanization did not affect taxonomical and functional alpha diversity, while it increased phylogenetic diversity. This suggests an increase in phylogenetic diversity in areas with a high degree of urbanization. It may result from the occurrence of non-native species, including also cultivated garden plants escaping into urban greenery (for example, trees), representing phylogenetic lineages absent in the native species pool. Moreover, urbanization causes disturbances in soil moisture and fertility, so more micro-niches within the community can be filled with phylogenetically distant species with different requirements. Williams et al. (2009) predicted that urbanization would result in increased phylogenetic diversity of the flora because of the colonization of novel city habitats by alien species. Plant species in urban grasslands, parks, and housing estates were similar in height, specific leaf area (SLA), and seed mass. Moreover, Ellenberg’s ecological indicators for light and temperature were also similar in three habitat types, except Ellenberg’s ecological indicators for soil moisture, soil reaction, and soil fertility, which were significantly higher in housing estates and parks than in urban grassland plant communities.

Our study demonstrated that the number of alien plant species had bell shape effect on plant species richness, alpha diversity, and functional richness, and functional dispersion. Thus, alien species decrease the alpha diversity of native plants, which is related to decreasing overall species pool, and dissimilarity among study plots. Moreover, the effect of alien plant species richness was stronger in urban grasslands and urban parks than in greenery around housing estates. Similar results were found for alien plant species cover, where species richness decreased with an increase in the cover of alien species for urban parks and grasslands where the slope for the housing estate starting increased with an increase in alien plant species cover and decreased in the highest range of predictor. However, the Shannon–Wiener diversity index increased with an increase in alien plant species cover in all three habitat types studied. Therefore, further studies should provide a detailed assessment of the impact of particular alien species on biodiversity and ecosystem services. In detail, as the role of invasive species is context-dependent (Czortek et al. 2023), their influence is not always straightforward. Moreover, alien species differ in residence time, which affects the level of their biological novelty (Schittko et al. 2020), and some of them, especially species naturalized before the sixteenth century, comprise a persistent part of plant communities (Mucina et al. 2016).

The community weighed means of SLA, seed mass, and height were similar in three habitat types and were not affected by urbanization. Urban plant communities influenced by specific environmental conditions and human pressure are more similar in three habitat types than we expected. Two of Ellenberg’s ecological indicators, i.e., soil moisture and fertility, as well as the vegetation height, significantly differed between studied habitat types. The results did not indicate differences in taxonomic, functional, or phylogenetic diversity between habitat types, suggesting that urbanization plays an important role in shaping the city flora.

We revealed that urbanization affects beta diversity, but the effect on biotic homogenization is habitat-dependent, and not both components of beta diversity, i.e., nestedness and turnover, react differently. Our results showed that in the three habitat types, the species turnover is higher than species nestedness. These results suggest that plant composition in urban green areas consists of randomly selected plant species rather than a fixed group. Moreover, higher functional and phylogenetic nestedness points to the importance of functional traits of plants connected with the phylogenetic in plant composition. We found that the difference in urbanization decreased taxonomic and phylogenetic nestedness in three habitats, whereas the effects of urbanization were higher in the housing estates. The functional nestedness decreased with the differences in urbanization in grassland and housing estates but increased in urban parks. This might be related to the more filtered species pool of urban parks, where an increase in urbanization can increase microhabitat diversity, related to expanding the size of the species pool core, responsible for nestedness. The taxonomic, phylogenetic, and functional species turnover increased with the urbanization difference in housing estates. In urban parks and grasslands, taxonomic and phylogenetic species turnover increased with the urbanization difference, but functional turnover decreased. This result suggests that human pressure filtering the functional plant composition increases dissimilarity between sites that differ by urbanization in urban parks. That result can be explained by the relatively high contribution of nestedness into overall functional beta diversity, compared with phylogenetic and taxonomic beta diversity. This might reflect the high significance of functional filtering in community assembly processes. Overall taxonomic, functional, and phylogenetic beta diversity increased with the urbanization difference in urban parks. In housing estates, taxonomic and phylogenetic beta diversity increased, but functional beta diversity decreased with the urbanization difference. In grassland, taxonomic and phylogenetic beta diversity decreased in the urbanization differences, but functional beta diversity increased.

We showed that urbanization strongly determined the beta diversity in housing estates compared with grasslands and urban parks. The greenery around housing estates is characterized by different management practices. Some parts of the vegetation are occasionally mowed where the species pool can be more filtered than in urban parks or grasslands. Moreover, various human activities on this greenery, like walking the dogs and trampling, may differ within housing estates located in different parts of the cities and compared with urban parks and grasslands. Thus, we observed a higher line trend in housing estates compared with urban parks and grasslands.

Our study indicated that urbanization leads to the similarity of city flora on three levels: taxonomical, functional, and phylogenic. In order to have a better understanding of the human pressure on urban vegetation, it should be considered more than taxonomical alpha and beta diversity. Using the functional traits and phylogenetic context allows for a better understanding of the plant species filtering process and the community assembly in the urban environment. Previous studies confirm that landscape change affects flora persistence (Williams et al. 2009; Andrade et al. 2015), and they suggest that the effects of urbanization extend throughout the functional and phylogenic diversity of plants.

In conclusion, our results suggest that human pressure in the city leads to similarities in the urban flora, where plant species with specific functional traits adapted to the urban environment. To increase heterogeneity in urban vegetation, urban planners should consider not only creating diverse green spaces but also eliminating alien plants. It is advisable to maximize the role of urban land management in providing a variety of habitats and promoting the wildness of plant biodiversity in cities to achieve sustainable urbanization.

## Supplementary Information

Below is the link to the electronic supplementary material.

Supplementary file1 (DOCX 181 KB)

## Data Availability

Data will be available on request.
