# Supplementary material for: How do urbanization and alien species affect the plant taxonomic, functional, and phylogenetic diversity in different types of urban green areas?
Source: Environ Sci Pollut Res Int. 2023 Jul 25;30(40):92390–403. doi: 10.1007/s11356-023-28808-y (PMC10447280; doi:10.1007/s11356-023-28808-y)
Supplement: Supplementary file 1 — Supplementary file1 (DOCX 181 KB) [file 11356_2023_28808_MOESM1_ESM.docx]

**Supplementary Materials**

**How do urbanization and alien species affect the plant taxonomic, functional, and phylogenetic diversity in different types of urban green areas?**

Łukasz Dylewski^1*^, Weronika Banaszak-Cibicka^1^, Łukasz Maćkowiak^2^, Marcin K. Dyderski^3^

1 Poznań University of Life Sciences, Department of Zoology, Wojska Polskiego 71C, 60-625, Poznań, Poland

2 Independent Researcher, Dmowskiego 81, 60-204 Poznań

3 Institute of Dendrology, Polish Academy of Sciences, Parkowa 5, 62-035, Kórnik, Poland

^*^corresponding author: lukasz.dylewski@up.poznan.pl

Tab. S1

Fig. S1

Tab. S1 The results non-metric multidimensional scaling of vegetation species composition.

| Variable | abbreviation | NMDS1 | NMDS2 | R^2^ | p |
| --- | --- | --- | --- | --- | --- |
| Imprevious surface area | ISA | -0.5693 | 0.8222 | 0.106 | 0.074 |
| Faith's phylogenetic diversity | PD | -0.9185 | 0.3954 | 0.352 | 0.001 |
| Mean pairwise distance | MPD | -0.6010 | -0.7993 | 0.041 | 0.376 |
| Mean nearest taxon distance | MNTD | -0.9660 | 0.2585 | 0.089 | 0.126 |
| Ellenberg EIV for light | EIV.L | 0.0014 | -1.0000 | 0.379 | 0.001 |
| Ellenberg EIV for temperature | EIV.T | 0.1285 | -0.9917 | 0.022 | 0.604 |
| Ellenberg EIV for soil moisture | EIV.M | 0.3904 | 0.9207 | 0.628 | 0.001 |
| Ellenberg EIV for soil reaction | EIV.SR | 0.5899 | 0.8074 | 0.526 | 0.001 |
| Ellenberg EIV for soil fertility | EIV.N | 0.7057 | 0.7085 | 0.719 | 0.001 |
| CWM of specific leaf area | SLA | 0.6945 | 0.7195 | 0.187 | 0.008 |
| CWM of seed mass | SM | -0.7767 | 0.6298 | 0.076 | 0.181 |
| CWM of height | H | -0.0621 | 0.9981 | 0.202 | 0.015 |
| Functional richness | FRic | -0.8233 | 0.5676 | 0.486 | 0.001 |
| Functional dispersions | FDis | -0.7434 | 0.6689 | 0.269 | 0.002 |
| Functional divergence | FDiv | -0.1175 | -0.9931 | 0.117 | 0.073 |
| Functional evenness | FEve | -0.9821 | 0.1885 | 0.097 | 0.123 |
| Species richness | sprich | -0.8798 | 0.4754 | 0.661 | 0.001 |


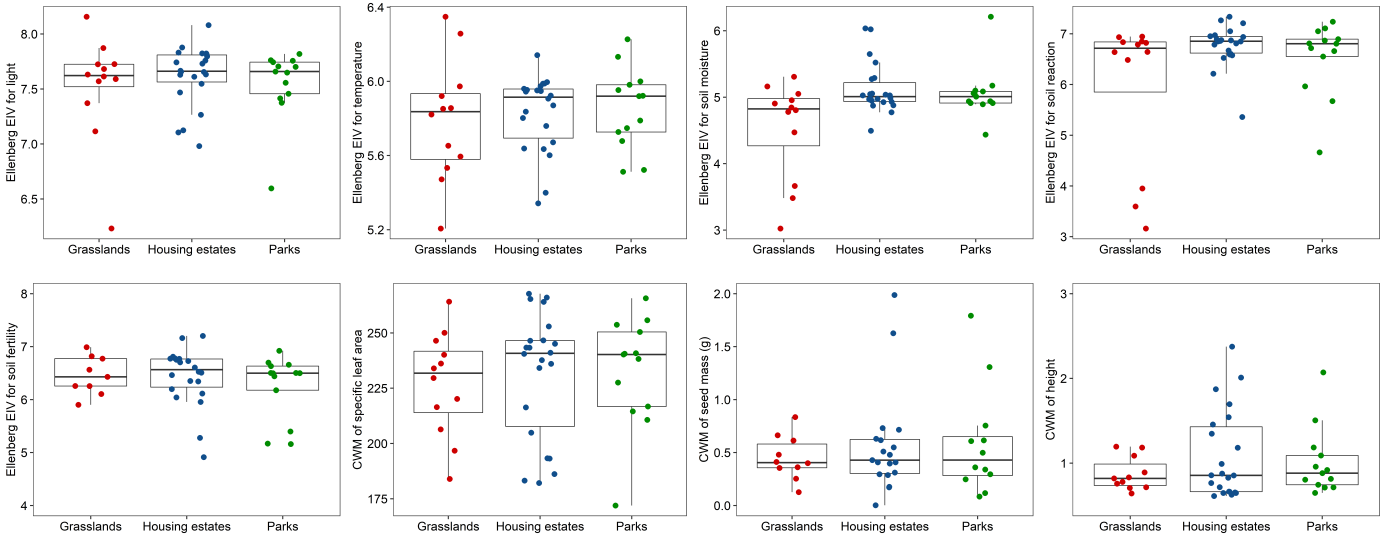


Fig. S1 Boxplot of Ellenberg EIV for light, temperature, soil moisture, soil reaction for soil fertility and community-weighted mean values of specific leaf area, of seed mass, of height for plant species in urban parks, housing estates and urban grasslands. The points represent a single observation.
